# Small cell lung cancer with thyroid gland oligometastasis: A case report

**DOI:** 10.1111/1759-7714.13740

**Published:** 2020-12-14

**Authors:** Toshiharu Tsutsui, Haruna Yamaki, Takashi Kumagai, Chisa Omori, Hiroaki Kobayashi, Yumiko Kakizaki, Yoshihiro Miyashita

**Affiliations:** ^1^ Lung Cancer and Respiratory Disease Center Yamanashi Prefectural Central Hospital Kofu Japan

**Keywords:** Oligometastasis, small cell lung cancer, thyroid metastasis

## Abstract

Extensive disease small cell lung cancer (ED‐SCLC) is a systemic disease characterized by diffuse metastases and a poor prognosis. Oligometastatic cases in ED‐SCLC are rare. This study reports the case of a 72‐year‐old Japanese male. A mass lesion was identified on chest computed tomography (CT). Fluorodeoxyglucose‐positron emission tomography/computed tomography (FDG‐PET/CT) revealed a solitary thyroid gland lesion with high FDG uptake as an extrapulmonary finding, suggesting thyroid cancer or a goiter. Upon confirmation of diagnosis, treatment of SCLC was prioritized, and chemoradiotherapy for limited disease SCLC was initiated without further examination of the thyroid gland. The thyroid nodule disappeared after treatment. Two years later, the disease recurred, and a thyroid nodule was found to have reappeared. Upon fine needle aspiration cytology of the thyroid, small cell carcinoma was detected. Therefore, in cases of SCLC, it is necessary to carefully investigate the thyroid for solitary lesions to consider the possibility of oligometastasis.

**Key points:**

**Significant findings of the study:**

Manifesting as a solitary lesion, oligometastasis, particularly in the thyroid, is rare in cases of ED‐SCLC.

**What this study adds:**

In SCLC cases, it is necessary to carefully investigate the thyroid for solitary lesions to consider the possibility of oligometastasis.

## Graphical abstract

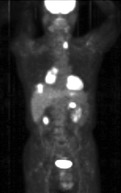

Oligometastasic cases in small cell lung cancer are rare, however, it is necessary to investigate the suspected lesions to consider the possibility of oligometastasis.

## Introduction

Small cell lung cancer (SCLC) is an aggressive disease characterized by diffuse metastases and a poor prognosis.^1^, ^2^ Approximately 60%–70% of newly diagnosed SCLC cases are reported to be extensive disease (ED). The standard treatment for ED‐SCLC is systemic chemotherapy, and the median survival is 8–13 months.^3^, ^4^

Defined by Hellman *et al*. as a transitional state between the localized and polymetastatic states, oligometastasis is the state most favorable for local therapy to impact long‐term survival.^5^ Recovery rates from oligometastasis have been confirmed in locally treated cases of colorectal cancer as well as in non‐SCLC patients.^6^, ^7^ However, since ED‐SCLC is a systemic disease, oligometastatic cases are rare. Xu *et al*. reported the efficacy of thoracic radiotherapy in oligometastatic ED‐SCLC.^8^ In this study, 78 of 270 patients (28.9%) had oligometastases. Shirasawa *et al*. also reported prognostic differences between oligometastatic and polymetastatic ED‐SCLC.^9^ A total of 49 of the 141 patients in this study were reported to have oligometastatic ED‐SCLC. Whilst there are studies which have reported oligometastatic ED‐SCLC, case reports for patients with this disease are rare.

Although the thyroid gland has a rich vascular supply, carcinomas rarely metastasize to the thyroid.^10^ Among the lung carcinomas metastasizing to the thyroid, adenocarcinomas are the most common.^11^ Meanwhile, small and large cell carcinomas rarely metastasize to the thyroid.^12^ This study reported a rare case of ED‐SCLC with thyroid oligometastasis.

## Case report

**Figure 1:**
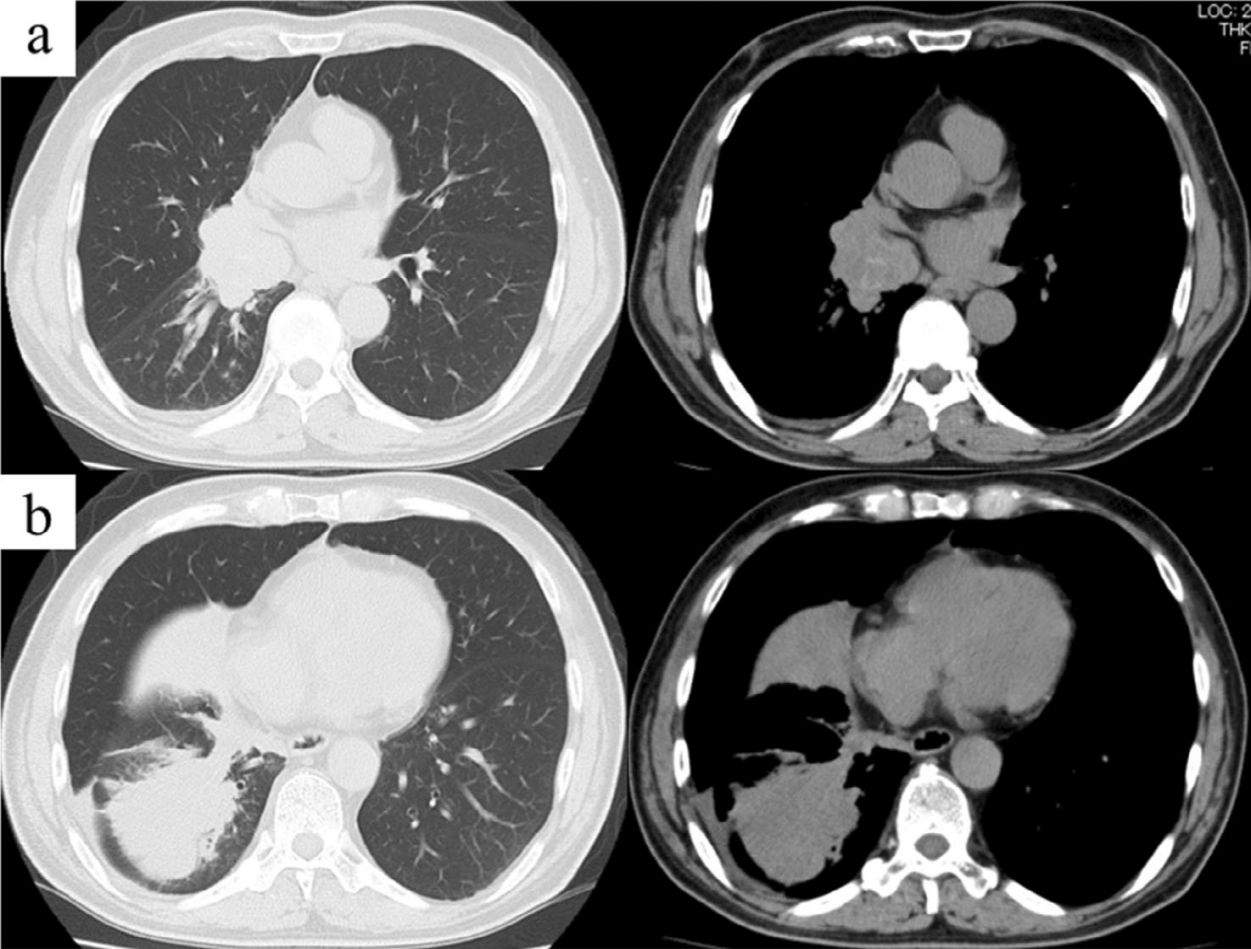
Chest plain computed tomography on the first visit revealed (**a**) right hilar lymphadenopathy and (**b**) a mass lesion measuring 70 mm in the right lower lobe.

**Figure 2:**
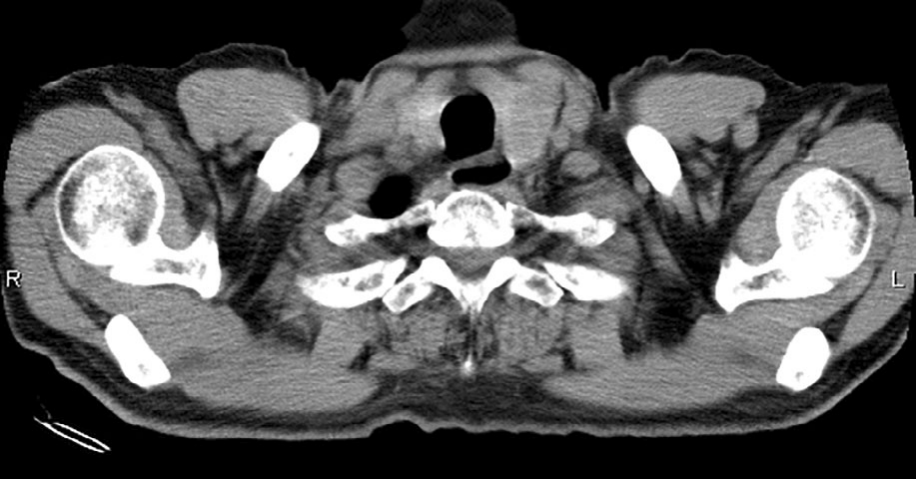
Chest plain computed tomography revealed a round low‐density area in the left thyroid lobe.

**Figure 3:**
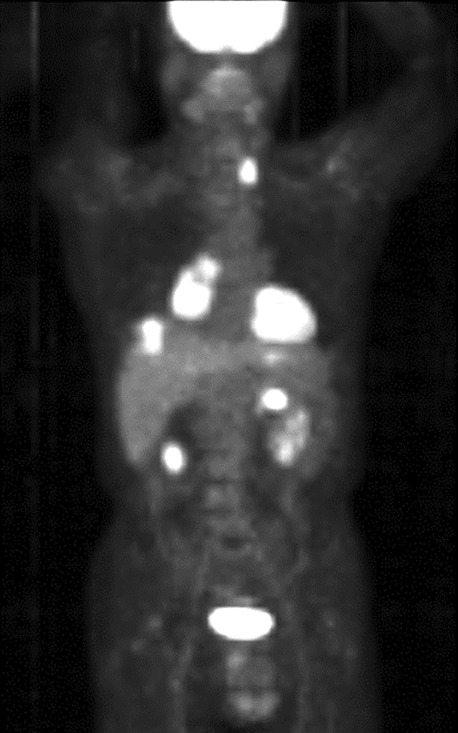
Fluorodeoxyglucose (FDG)‐positron emission tomography revealed high FDG uptake in the left thyroid lobe and the ascending colon as extrapulmonary findings.

**Figure 4:**
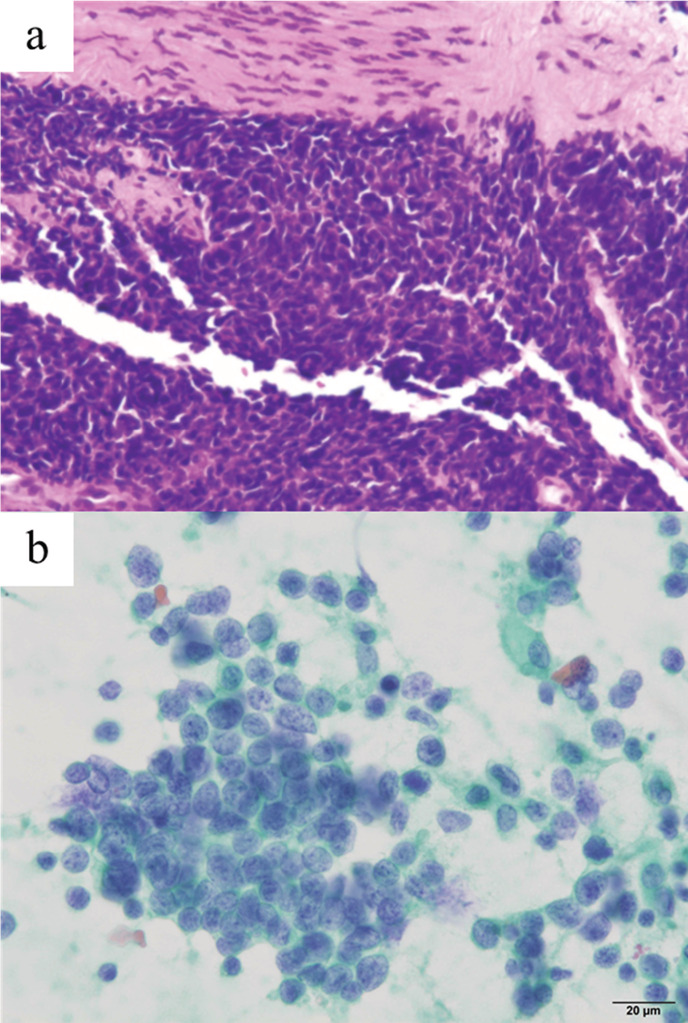
(**a**) Hematoxylin and eosin staining of transbronchial biopsy showed small cell lung cancer (×100 magnification). (**b**) Papanicolaou staining of thyroid fine needle aspiration biopsy (×400 magnification). Small cell carcinoma cells are characterized by high nuclear to cytoplasmic ratio and coarse chromatin.

A 72‐year‐old Japanese male visited our hospital with a complaint of persistent cough. He was a current smoker with a past history of nephrolithiasis and hyperuricemia. A mass lesion with hilar and mediastinal lymphadenopathy was identified in the right lower lobe and a round low‐density area in the left thyroid lobe on chest plain computed tomography (CT) (Fig 1, 2). Fluorodeoxyglucose‐positron emission tomography/computed tomography (FDG‐PET/CT) demonstrated high FDG uptake in the lung lesions. Furthermore, high FDG uptake was identified in the left thyroid lobe and ascending colon (Fig 3), for which the differential diagnoses were thyroid cancer or goiter, and colorectal cancer or metastatic tumor, respectively. Brain metastasis was not identified on magnetic resonance imaging (MRI). The patient was diagnosed with SCLC via transbronchial biopsy (Fig 4a). Colonoscopy was performed to consider the possibility of metastatic colorectal cancer; however, no abnormalities were found. A solitary lesion in the thyroid gland secondary to SCLC metastasis was not considered, so immediate treatment of SCLC was prioritized. Without further examining the thyroid, chemoradiotherapy and prophylactic cranial irradiation (PCI) for limited disease SCLC was performed. The response of the therapy was complete remission (CR). Consequently, the thyroid nodule disappeared after treatment. However, the thyroid nodule reappeared two years later. Thyroid ultrasonography revealed a diffuse heterogeneous lesion with an irregular margin. Fine needle aspiration cytology of the thyroid gland revealed small cell carcinoma and oligometastasis secondary to SCLC (Fig 4b). High FDG uptake was again identified in the left thyroid lobe and the right hilum of the lung. We determined local recurrence due to the concordance of lesions at the initial diagnosis. Chemotherapy was initiated and the pulmonary and thyroid lesions disappeared. Since his treatment, the patient has not experienced an episode of recurrence for four years, and remains healthy.

## Discussion

Thyroid incidentalomas are frequently identified via whole body screening using FDG‐PET/CT. Among 128 patients, Makis *et al*. identified FDG positive focal thyroid incidentalomas in 2.2% of oncologic PET/CT scans, 10.9% of which were malignant.^13^ Furthermore, there was a significant overlap between the ranges of benign and malignant thyroid incidentalomas with benign maximum standardized uptake value (SUVmax) values ranging from 2.1 to 30.5, and malignant SUVmax values ranging from 3.4 to 28.1. Thus differentiation between benign and malignant thyroid incidentalomas using only SUVmax was difficult. The SUVmax of this patient's thyroid uptake was 7.19, so thyroid cancer and goiter were possible. Due to its low malignancy rate and rare occurrence, oligometastatic thyroid cancer was not considered. Even if the lesion was due to primary thyroid cancer, SCLC treatment was prioritized. If thyroid oligometastasis had been diagnosed at the time of initial therapy, addition of local therapy for thyroid lesion via radiation may have been considered.

Thyroid metastasis is a rare sequela. In an autopsy study of advanced cancer, the rate of metastasis to the thyroid gland was 8.6%. Shimaoka *et al*. reported thyroid metastasis in 81 out of 2646 cases (3.1%).^14^ According to Willis, low rates of thyroid metastasis were due to the increased blood flow which prevented malignant cells from adhering to thyroid tissue, and high iodine and oxygen concentrations which inhibited malignant cell proliferation.^15^

Differences in survival rates between oligometastatic and polymetastatic ED‐SCLC remain unclear. Shirasawa *et al*. reported a better prognosis for oligometastatic ED‐SCLC than polymetastatic disease.^9^ Furthermore, the study suggested that local brain irradiation may benefit patients with oligometastatic ED‐SCLC. The patient in this case study also achieved long‐term survival, affirming the previous report.

We herein report a rare case of ED‐SCLC with thyroid gland oligometastasis. In cases of SCLC, it is necessary to consider the rare possibility of thyroid oligometastasis, and solitary lesions must be carefully evaluated.

## Disclosure

The authors report no conflict of interest.
